# Comparison of anal dilatation versus no dilatation with a standardized dilator in reducing pain after hemorrhoidectomy: a randomized control trial

**DOI:** 10.1186/s12876-022-02409-4

**Published:** 2022-08-09

**Authors:** Imam Sofii, Handy Darmawan, Amelia Sophia Ramadhini, Fauzan Kurniawan, Ahmad Shafa Hanif

**Affiliations:** Digestive Surgery Division, Department of Surgery, Faculty of Medicine, Gadjah Mada University/Dr, Sardjito Hospital, Jl. Kesehatan No. 1, Yogyakarta, 55281 Indonesia

**Keywords:** Dilatation, Fecal incontinence, Hemorrhoid, Hemorrhoidectomy, Pain management

## Abstract

**Background:**

Post-operative pain is the main problem of hemorrhoidectomy. An adequate pain management can promote early mobilization, fast recovery, and reduce hospitalization costs. This study aimed to investigate the role of preoperative anal dilatation using a standardized anal dilator in reducing post-operative pain.

**Method:**

This study was conducted using randomized prospective trial with a total of 40 subjects, who were divided into 2 groups. The first group received preoperative anal dilatation using a 33 mm anal dilator for 20 min, while the second group did not. The post-operative anal pain, edema, bleeding, and incontinence were observed in the first, second, and seventh day.

**Result:**

The post-operative pain was significantly lower in the preoperative anal dilatation group for all days of observation (*p* < 0.05). The difference of post-operative bleeding and edema between groups were not significant. Fecal incontinence was initially significantly higher in the preoperative anal dilatation group, but the difference was insignificant at the seventh day (*p* = 0.500).

**Conclusion:**

Preoperative anal dilatation significantly reduced post-operative pain. The side effect of fecal incontinence was only temporary until the seventh day after surgery.

*Trial Registration* This trial was registered on Thai Clinical Trials Registry (TCTR) with TCTR identification number TCTR20220314002, on 14/03/2022 (retrospectively registered).

## Highlights

- Pain is a common complication in patients after undergoing hemorrhoidectomy.
- This study found preoperative anal dilatation significantly reduced post-operative pain.

## Background

Post-operative pain is one of the main issues of hemorrhoidectomy and remains a distressing problem, for patients and physicians. A sufficient pain management can lead to higher satisfaction, earlier mobilization, faster recovery, and lower health care cost [1]. Several methods have been described in combination with conventional hemorrhoidectomy to reduce pain. Several studies were using pharmacological approaches and minimal invasive methods to reduce post-operative pain [2–4]. In this study, a simple method was done using an anal dilatator to reduce post-operative pain.

Anal dilatation was first described by Lord PH to treat hemorrhoids and anal fissures using six fingers [5, 6]. However, anal dilatation alone often results in relapse of symptoms and other complications including fecal incontinence [7]. In this study, we modified the original Lord’s anal dilatation procedure using a dilator, followed by hemorrhoidectomy. Furthermore, post-operative pain, edema, and fecal incontinence were observed after performing hemorrhoidectomy.

## Methods

This was a randomized prospective trial using a total of 40 subjects. A total sampling with convenience sampling was used to determine sample size. The current study was performed between January and December 2021. Patients with a 3^rd^ grade hemorrhoid with 2 to 3 piles were selected. However, patients with preoperative fecal incontinence, history of colorectal cancer, anal fissure, colitis, previous anorectal bleeding, previous hemorrhoidectomy, hemorrhoid with thrombus, and other anorectal surgery were excluded from the study. The subjects were randomly assigned into two groups using simple randomization technique.

**Fig. 1:**
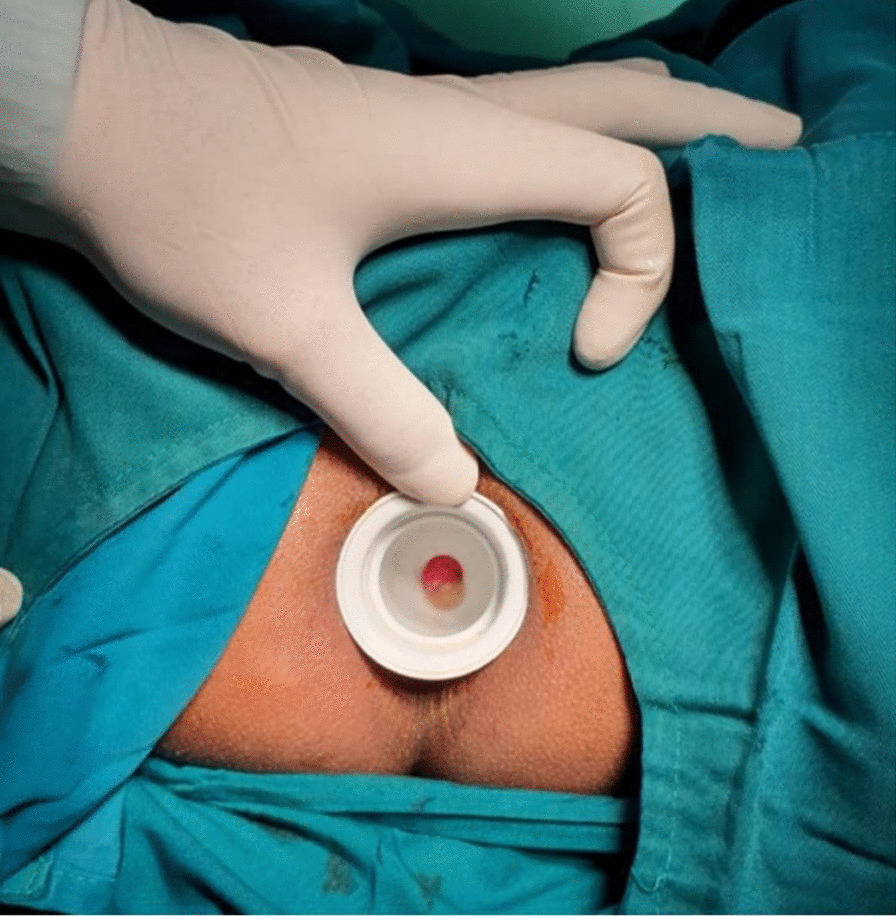
Dilatation before hemorrhoidectomy procedure with standardized dilator

Anal dilatation was performed before surgery using a dilator with diameter of 33 mm. Dilator was lubricated with water based sterile gel before inserted into anal canal. After anesthesia agent was administered and the patient was relaxed, the dilator was gently inserted into anal canal and rectum (Fig. 1). The dilatation process was maintained for 20 min. This procedure was performed for the first group, while the second group did not receive the anal dilatation procedure. Furthermore, the procedure was followed by hemorrhoidectomy. The hemorrhoid bundle was first clamped and tensed to visualize the mucocutaneous junction. It was then excised using a scalpel and its apex was ligated. The mucosal wound was sutured using a multifilament absorbable suture to the anal verge in a running locking fashion.

The post-operative anal pain, edema, bleeding, and incontinence were evaluated in the first, second, and seventh day after surgery. Post-operative anal pain was evaluated using the Visual Analog Scale (VAS), categorized as mild pain (VAS 1–4), moderate pain (VAS 5–6), and severe pain (VAS 7–10) [8]. We used single blind examination by examining the pain scale without knowing any dilatation. In addition, both groups received the same post-operative analgesic using 30 mg of intravenous ketorolac every 8 h for the first 24 h, which was subsequently switched to oral 500 mg mefenamic acid every 8 h until the third day.

The procedure of surgical hemorrhoidectomy and the postoperative care were performed based of the Clinical Pathway published by The Indonesian Surgeon Collage. This study was approved by the Institutional Review Board of the Faculty of Medicine, Public Health and Nursing, Gadjah Mada University/Dr. Sardjito General Hospital, Yogyakarta, Indonesia, Ref. No: KE/FK/0083/EC/2022 according to the Declaration of Helsinki. This trial was registered on Thai Clinical Trials Registry (TCTR) with TCTR identification number TCTR20220314002, on 14/03/2022 (retrospectively registered).

Baseline characteristics of the patients are presented in Table 1. There is no change on trial outcome after the trial commenced, because all participants had finished the study protocol. In the bivariate analysis, differences between the anal dilatation and non-anal dilatation groups in the first, second, and seventh days after surgery were presented on Table 2. The variables were analyzed using chi-square tests. The results are considered to be significant if *p* < 0.05. Multivariate analysis of the variables was calculated using logistic regression (Table 3). Statistical review of the study was performed by a biomedical statistician. The data were analyzed using SPSS (IBM Corp., Armonk, NY) software. The report of this trial had been written according to the CONSORT 2010 guideline [9].

**Table 1:** Baseline characteristics of the patients

Variables	N (%)	Mean ± SD	Median (min–max)
Sex	Male: 16 (40)		
	Female: 24 (60)		
Age		43.25 ± 1,289	45 (25–57)
Post-operative day 1			
Anal bleeding	Yes: 1 (2,5)		
	No: 39 (97.5)		
Anal pain	Severe: 2 (5)		
	Moderate: 9 (22.5)		
	Mild: 25 (62.5)		
	No Pain: 4 (10)		
Anal oedema	Yes: 22 (55)		
	No: 18 (45)		
Fecal incontinence	Yes: 22 (55)		
	No: 18 (45)		
Post-operative day 2			
Anal bleeding	Yes: 1 (2,5)		
	No: 39 (97.5)		
Anal pain	Severe: 0 (0)		
	Moderate: 0 (0)		
	Mild: 22 (55)		
	No Pain: 16 (45)		
Anal oedema	Yes: 9 (22.5)		
	No: 31 (77.5)		
Fecal incontinence	Yes: 19 (47.5)		
	No: 21 (52.5)		
Post-operative day 7			
Anal bleeding	Yes: 1 (2.5)		
	No: 39 (97.5)		
Anal pain	Severe: 0 (0)		
	Moderate: 0 (0)		
	Mild: 11 (27.5)		
	No Pain: 29 (72.5)		
Anal oedema	Yes: 3 (7.5)		
	No: 37 (92.5)		
Fecal incontinence	Yes: 1 (2.5)		
	No: 39 (97.5)

**Table 2:** Bivariate Analysis of anal dilatation versus no anal dilatation

Variables		Dilatation	No Dilatation	*p-*value
Post-operative day 1				
Anal oedema	Yes	10	12	0.525
	No	10	8	
Anal pain	No pain	4	0	< 0.001*
	Mild	16	9	
	Moderate	0	9	
	Severe	0	2	
Anal bleeding	Yes	0	1	0.500
	No	20	19	
Fecal incontinence	Yes	15	7	0.011*
	No	5	13	
Post-operative Day 2				
Anal oedema	Yes	4	16	0.500
	No	15	5	
Anal pain	No pain	14	4	0.004*
	Mild	6	16	
	Moderate	0	0	
	Severe	0	0	
Anal bleeding	Yes	0	1	0.500
	No	20	19	
Fecal incontinence	Yes	14	5	0.004*
	No	6	15	
Post-operative day 7				
Anal oedema	Yes	1	2	0.500
	No	19	18	
Anal pain	No pain	18	11	0.031*
	Mild	2	9	
	Moderate	0	0	
	Severe	0	0	
Anal bleeding	Yes	0	1	0.500
	No	20	19	
Fecal incontinence	Yes	1	0	0.500
	No	19	20	

Datas with p value below 0.05 (asterisk) are significance

Bold highlights the significant data value

**Table 3:** Multivariate analysis using logistic regression among independent variables

Day	Variables	R Square	*p-*Value
1	Anal bleeding	0.469	0.143
	Anal pain		< 0.001*
	Anal oedema		0.265
	Fecal incontinence		0.209
2	Anal bleeding	0.372	0.642
	Anal pain		0.005*
	Anal oedema		0.646
	Fecal incontinence		0.018*
7	Anal bleeding	0.180	0.784
	Anal pain		0.030*
	Anal oedema		0.572
	Fecal incontinence		0.431

Datas with p value below 0.05 (asterisk) are significance

Bold highlights the significant data value

## Results

Baseline characteristics of the subjects showed no significant difference in age, sex, and body mass index (BMI) between dilatation and no dilatation groups (Table 1).

The post-operative pain was significantly lower in preoperative anal dilatation group with *p*-values of all days of observation < 0.05 (Table 2).

There was only one post-operative bleeding observed in this study, which was found in the group without preoperative anal dilatation (*p*-value > 0.05). The post-operative edema was lower in the preoperative anal dilatation group of all days of observation, which were statistically insignificant with *p*-value > 0.05. In addition, fecal incontinence was higher in the preoperative anal dilatation group with *p* values 0.011 and 0.004 for the first and second day, respectively. However, the result of the seventh day showed insignificant result with *p*-value 0.500. In addition, the severity of fecal incontinence found in this study was only minor with flatus and liquid stool incontinence.

Multivariate analysis showed that on the first day of observation, preoperative anal dilatation contributed 46.9% for the reduction of anal pain with *p*-value < 0.001 (Table 3).

On the second day of observation, preoperative anal dilatation contributed 37.2% for the reduction of anal pain and the occurrence of fecal incontinence with *p*-values 0.005 and 0.018, respectively. In addition, on the last day of observation, preoperative anal dilatation contributed 18% for the reduction of anal pain.

## Discussion

The majority of hemorrhoid cases, especially for the third degree and above, were surgically treated [10]. The pain after surgery is still the main problem for the patients. Extensive anoderm excision might cause anal spasm, which subsequently cause pain [11]. There were several published methods for reducing pain after hemorrhoidectomy [1, 12]. Previous study used flavonoids and metronidazole, which resulted in a reduction of pain after excisional hemorrhoidectomy [12]. Opioid analgesics were also used to reduce pain after hemorrhoidectomy [3]. In this study, we used a simple non-pharmacological method to reduce pain after hemorrhoidectomy, which showed a significant result.

The reduction of post-operative pain might be due to reduced anal sphincter contraction. Relaxed anal sphincter reduced the risk of anal spasm and subsequently reduced post-operative pain [10]. The dilatation was commonly performed using fingers (Fig. 1), which are different in size and power among physicians or surgeons [5, 6]. The use of anal dilator for reducing anal spasm and pain after hemorrhoidectomy had been proposed to be performed post-operatively [13]. However, the patient can still experience pain during the procedure. In contrast, our method was performed preoperatively under anesthesia with a standardized 33 mm anal dilator, which is painless, simpler, effective, and reproducible.

The only significant side effect found in this study was fecal incontinence. The stretched anal sphincter is weakened in function and subsequently caused fecal incontinence [13]. However, the incontinence found in this study was only minor with flatus and liquid stool incontinence and was proved to be temporary. The majority of cases resolved within 7 days after surgery. It might be due to the use of a standardized dilator, which is capable of preventing either over stretch or injured anal sphincter. Previous study proposed the use of internal sphincterotomy for reducing anal spasm after hemorrhoidectomy and subsequently reduced post-operative pain [14]. However, this method was reported to cause both urinary retention and fecal incontinence. In contrast, our method had less complication of temporary fecal incontinence.

Anal dilatation was also reported to reduced pain in other anorectal surgeries. The study about anal dilatation in perianal fissure also showed significant reduction in pain and fecal incontinence as its temporary short-term complication [15]. The side effect of fecal incontinence can be reduced by smoking cessation, low sodium diet, caffeine restriction, and high fiber diet [16]. Therefore, with careful education about its temporary side effect, this method can be an effective option for reducing pain after hemorrhoidectomy.

## Conclusions

Preoperative anal dilatation significantly reduced pain after hemorrhoidectomy. Post-operative bleeding and edema were not significantly reduced by preoperative anal dilatation. The side effect of fecal incontinence was only temporary until the seventh day after surgery.

## Data Availability

The protocol and datasets generated during and/or analyzed during the current study are available from the corresponding author on reasonable request.
